# Abscesses

**Published:** 1874-05

**Authors:** 


*Abscesses.*—The popular method of removing pus from the cavity of an abscess is by means of the aspirator. As a means of diagnosis, or a means for the simple removal of the fluid from a cavity, it is one of the most successful and convenient. As a curative measure it is not regarded so favorably. The simple removal of the fluid in many cases is not sufficient to prevent its return, and it becomes necessary to lay the walls open by free incision; secure a drainage by seton, and cause the cavity to heal from the bottom.

